# Development and validation of a simple screening tool for caregiver grief in dementia caregiving

**DOI:** 10.1186/s12877-019-1070-x

**Published:** 2019-02-27

**Authors:** Tau Ming Liew, Bee Choo Tai, Philip Yap, Gerald Choon-Huat Koh

**Affiliations:** 1 Department of Geriatric Psychiatry, Institute of Mental Health, 10 Buangkok View, Singapore, 539747 Singapore; 2 Psychotherapy Service, Institute of Mental Health, Singapore, Singapore; 3 Saw Swee Hock School of Public Health, National University of Singapore, Singapore, Singapore; 4 Department of Geriatric Medicine, Khoo Teck Puat Hospital, Singapore, Singapore; 5 Geriatric Education and Research Institute, Singapore, Singapore

**Keywords:** Dementia, Caregiver grief, Screening tool, Marwit-Meuser caregiver grief inventory

## Abstract

**Background:**

Loss and grief are experienced by caregivers of persons with dementia (PWD), relating to the ambiguous loss of PWD even when they are still alive and the anticipation of future loss related to their physical death. Such experience of caregiver grief is not easily recognized in clinical practice, despite its association with adverse effects such as caregiver burden, caregiver depression and caregivers’ desire to place the PWD in nursing homes. We constructed a simple screening tool – based on factors associated with caregiver grief – to identify caregivers with high grief.

**Methods:**

Spouses or children of community-dwelling PWD (*n* = 403) completed self-administered questionnaires containing a well-established grief scale and information related to the caregiver and PWD. We split the study sample into two – the derivation sample (*n* = 300) was used to identify factors associated with grief (using logistic regression) and derive a simple tool based on the number of identified factors; the validation sample (*n* = 103) evaluated the performance of the tool using the receiver-operating-characteristic-curve-analysis (ROC).

**Results:**

Four key factors were identified by the multivariable regression – more severe dementia (odds ratio, OR 6.9), behavioral problems in PWD (OR 5.0), spousal caregivers (OR 6.0) and daily caregiving (OR 3.0). The screening tool (based on the number of key factors) had an area under ROC of 0.77. At the optimal cut-off of ≥2 key factors, the tool had a sensitivity of 0.91 and a specificity of 0.42.

**Conclusions:**

The identified factors are consistent with current understanding on caregiver grief. They can be easily integrated into the workflow of routine services to screen for caregivers who are more likely to benefit from further grief-related assessment.

**Electronic supplementary material:**

The online version of this article (10.1186/s12877-019-1070-x) contains supplementary material, which is available to authorized users.

## Background

Bereavement in dementia caregiving can occur long before the physical death of the PWD. The PWD, despite being physically present, becomes increasingly disconnected from the caregiver with worsening of cognitive decline [1]. This ambiguous loss, as well as the anticipation of future loss related to the physical death of PWD, engender the experience of loss and grief in caregivers in the pre-death context (henceforth referred to as ‘caregiver grief’) [1]. Such experience of caregiver grief has been highlighted as a key challenge faced by family caregivers in a recent systematic review, with the experience of loss and grief accentuating caregivers’ unmet needs for companionship, nurturance and emotional security [2]. In another systematic review, the theme of loss and grief was also described as central to the lived-in experience of spousal caregivers [3].

When the experience of loss and grief is not recognized, caregivers may have difficulty noticing the changes that have occurred in the PWD and may fight the inevitable disease progression in the PWD. As such, they may resort to control-based coping strategies – by becoming more authoritarian and paternalistic when relating to the PWD – which can often lead to the sense of loss of autonomy in the PWD and the feeling of helpless in the caregivers (over what they cannot control) [4, 5]. Consequently, caregiver grief has been linked to negative outcomes such as caregiver depression [6] and caregivers’ desire to institutionalize the PWD [7]. In a most recent longitudinal study [8], it has also been shown to predict caregiver depression after 2.5 years, independent of the well-established effect of caregiver burden.

Unfortunately, caregiver grief can be challenging to recognize in routine clinical practice. It is often disenfranchised (that is, not socially sanctioned) and hence may not be readily described by the caregivers [9]. It may also be missed by caregiver burden scales which are often used in dementia services [1]. Even while it is possible to detect caregiver grief with scales such as the 50-item Marwit-Meuser Caregiver Grief Inventory (MM-CGI) or its abridged 18-item MM-CGI-Short-Form [10, 11], the length of these scales may hinder their routine use in clinical practice which typically has high patient load. One alternative approach is to have a simple tool that can alert clinicians to high-risk caregivers who may then be further evaluated using specific grief scales such as MM-CGI, hence allowing more efficient use of clinical resources in the detection of caregiver grief.

In this study, we sought to construct such a simple screening tool – based on factors associated with caregiver grief – to aid in the identification of those at greater risk of high grief and may potentially benefit from further grief-related assessment.

## Methods

### Participants and procedures in the derivation sample

The derivation sample is based on an ongoing study on dementia caregiving at two tertiary hospitals in the North-East of Singapore. The details of the research have previously been described [1]. Briefly, 300 caregivers were recruited as they accompanied the PWD to the dementia care service of the two hospitals. We consecutively sampled the caregivers and had a response rate of 87.8% in the recruitment. Our inclusion criteria comprised: (i) spouse or child of PWD; (ii) caring for PWD who is residing in the community; (iii) able to read English; and (iv) age ≥ 21 years. At the point of recruitment, participants completed on-site a set of self-administered questionnaires comprising a caregiver grief scale (Marwit-Meuser Caregiver Grief Inventory) (MMCGI) [10] and information related to the caregiver and PWD. Ethical approval was obtained from the Domain Specific Review Board of Singapore.

### Measures

The Marwit-Meuser Caregiver Grief Inventory (MM-CGI) is among the few scales that measure caregiver grief [6, 9]. It was empirically-developed through extensive focus-group interviews with caregivers of PWD, with the aim of capturing the various aspects of losses experienced by caregivers [10]. MM-CGI includes 50 items which are self-rated by participants on 5-point Likert scales based on how much they agree with the statements, and summed to generate a total score ranging from 50 to 250 (with higher scores indicating higher levels of grief). In previous studies, total scores > 175 have been reported to indicate high levels of caregiver grief which may benefit from clinical attention and intervention [1, 10]. MM-CGI has previously been validated in our local population [1, 5, 8, 12, 13].

Apart from the MM-CGI, we also captured key information that characterize the caregiver (*age, gender, ethnicity, marital status, employment status, education, relationship with PWD, whether the caregiver stayed with the PWD, duration of caregiving, whether the caregiver provided daily care*, and *primary caregiving role*) and the PWD (*age, gender, duration of dementia diagnosis, diagnosis of dementia before 65 years old, stage of dementia*, and *presence of severe behavioral problems*). The stage of dementia was assessed based on the descriptors of the three dementia severities in the revised third edition of Diagnostic and Statistical Manual of Mental Disorders (DSM-III-R) [14]. Participants chose the description that best matched the PWD – still capable of independent living (mild stage), needs some assistance with daily living (moderate stage), or needs round-the-clock supervision (severe stage). This brief measure is consistent with the dementia severity descriptions in DSM-5 [15], and was previously shown to have adequate agreement with Clinical Dementia Rating Scale (kappa 0.56–0.6) [16, 17]. The presence of severe behavioral problem was indirectly measured through the need for admission to the geriatric psychiatry ward, indicating a behavioral problem that was too severe to be managed in the community setting.

### Constructing a simple screening tool

To construct a screening tool, we first performed bivariate analyses (using chi-square test for categorical variables and two-sample T-test for continuous variables) to identify factors associated with high grief in caregivers (defined as MM-CGI > 175). All variables with *p* ≤ 0.05 in the bivariate analyses were considered for inclusion in the multivariable logistic regression analysis. Variables with *p* > 0.05 in the multivariable regression were removed through backward variable selection method [18]. We then evaluated the goodness of fit and discriminative value of the final regression model, with further details available in Additional file 1.

Using the identified factors from the multivariable regression, we aimed to construct a simple screening tool that is convenient to use and can be easily applied in practice [18]. To that end, we evaluated whether the number of the identified factors alone could sufficiently be useful to detect caregivers with high grief. A summary score was calculated for all the participants, with each identified factor assigned one point. The overall ability of the tool to discriminate between caregivers with and without high grief was evaluated using the area under the receiver operating characteristics curve (AUROC), with AUROC of 0.7–0.8 is considered acceptable, and more than 0.8 is considered excellent [19].

### Evaluating the simple screening tool in an independent validation sample

We validated the screening tool in an independent sample, based on our separate study which recruited another 103 caregivers to complete the Chinese MM-CGI (instead of the English MM-CGI). The details of this other study are available in a separate publication [5]. Briefly, this other study was similar to the current study (sharing the same recruitment site, study period, inclusion criteria and study procedures), with the only difference in the language of administration (Chinese MM-CGI was administered in the separate study, in contrast to English MM-CGI which was administered in the current study). We have previously demonstrated the equivalence in MM-CGI scores between the English and Chinese version [5], and hence could use this other study (based on Chinese MM-CGI) as the validation sample. The calculations of AUROC was repeated in this validation sample to evaluate the discriminative ability of our screening tool. All statistical analyses were performed using STATA software version 14.

## Results

Of the 300 participants in the derivation sample, 265 of them were children caregivers and 35 were spousal caregivers. The participants had a mean MM-CGI score of 140.0 (standard deviation, SD 35.4). Eighteen per cent of them had high caregiver grief (with MM-CGI > 175). Table 1 shows the demographic information of the participants and their associations with high grief in bivariate analyses.

**Table 1:** Demographic characteristics of caregivers and persons with dementia in the derivation sample (*n* = 300), and the association with high caregiver grief ^a^

Variable	Overall sample (n = 300)	High grief ^a^ (*n* = 54)	Average or low grief (*n* = 246)	*P* value ^b^
Variables related to caregivers				
Age, mean (SD)	52.1 (11.0)	55.6 (12.3)	51.4 (10.5)	**0.010**
Male gender, n (%)	120 (40.0)	20 (37.0)	100 (40.7)	0.624
Ethnicity, n (%)				**0.006**
Chinese	247 (82.3)	37 (68.5)	210 (85.4)	
Malay	25 (8.3)	10 (18.5)	15 (6.1)	
Indian/Eurasian/Others	28 (9.3)	7 (13.0)	21 (8.5)	
Marital status, n (%)				**0.020**
Married	203 (67.7)	44 (81.5)	159 (64.6)	
Single	76 (25.3)	6 (11.1)	70 (28.5)	
Widowed/Divorced/Separated	21 (7.0)	4 (7.4)	17 (6.9)	
Employment status, n (%)				**0.013**
Working full-time	179 (59.7)	24 (44.4)	155 (63.0)	
Working part-time	36 (12.0)	6 (11.1)	30 (12.2)	
Not working	85 (28.3)	24 (44.4)	61 (24.8)	
Highest education, n (%)				**0.034**
Secondary or below	190 (63.3)	41 (75.9)	149 (60.6)	
Tertiary	110 (36.7)	13 (24.1)	97 (39.4)	
Relationship with the PWD, n (%)				**< 0.001**
Child	265 (88.3)	36 (66.7)	229 (93.1)	
Spouse	35 (11.7)	18 (33.3)	17 (6.9)	
Staying with the PWD, n (%)	199 (66.3)	42 (77.8)	157 (63.8)	**0.049**
Duration of caregiving in years, mean (SD)	6.6 (6.7)	5.5 (6.1)	6.8 (6.8)	0.197
Providing daily caregiving, n (%)	220 (73.3)	49 (90.7)	171 (69.5)	**0.001**
Primary caregiving role, n (%)	218 (72.7)	47 (87.0)	171 (69.5)	**0.009**
Variables related to PWD				
Age, mean (SD)	79.5 (8.1)	77.5 (8.6)	79.9 (8.0)	0.051
Male gender, n (%)	86 (28.7)	21 (38.9)	65 (26.4)	0.067
Duration of dementia diagnosis in years, mean (SD)	4.5 (3.5)	4.0 (2.9)	4.6 (3.6)	0.222
Diagnosis of dementia before 65 years old, n (%)	34 (11.3)	9 (16.7)	25 (10.2)	0.172
Stage of dementia, n (%)				**0.001**
Mild	49 (16.3)	2 (3.7)	47 (19.1)	
Moderate	127 (42.3)	18 (33.3)	109 (44.3)	
Severe	124 (41.3)	34 (63.0)	90 (36.6)	
Severe behavioral problem, n (%)	18 (6.0)	10 (18.5)	8 (3.3)	**< 0.001**

*SD* standard deviation, *PWD* persons with dementia, *MM-CGI* Marwit-Meuser Caregiver Grief Inventory

^a^High caregiver grief was defined as MMCGI> 175

^b^Test of difference between high versus average or low grief: chi-square test for categorical variables and two-sample T-test for continuous variables. Bold-faced *p* values are ≤0.05

The results from multivariable logistic regression are presented in Table 2. Only four variables remained significant in the multivariable regression, namely, stage of dementia, severe behavioral problems in PWD, spousal caregivers and caregivers who provide daily care. The final model demonstrated good model-fit and discriminative value (further results on the model evaluation are available in Additional file 2). Notably, it had an acceptable ability to discriminate caregivers with high grief, with an AUROC of 0.76 (95% CI 0.69–0.82, *p* < 0.001).

**Table 2:** Factors associated with high caregiver grief in the final model of the multivariable regression, based on the derivation sample (*n* = 300)

Variable	OR (95% CI)	P value
Stage of dementia		0.012
Mild	ref	
Moderate to severe ^a^	6.9 (1.5–31.4)	
Severe behavioral problem in PWD		0.003
No	ref	
Yes	5.0 (1.7–14.4)	
Relationship with PWD		< 0.001
Child	ref	
Spouse	6.0 (2.7–13.7)	
Providing daily caregiving		0.028
No	Ref	
Yes	3.0 (1.1–8.2)	

*OR* odds ratio, *95% CI* 95% confidence interval, *PWD* persons with dementia, *ref* reference group in the multivariable logistic regression, *MM-CGI* Marwit-Meuser Caregiver Grief Inventory

^a^Moderate and severe stage of dementia were grouped together so that the resulting OR had similar magnitude to those of the other three variables. This step enabled the subsequent construction of a brief screening tool based on equal weighting of the identified factors, which has the benefit of simplicity

Using the four key factors identified from multivariable regression, we evaluated whether a simple screening tool – based on the number of key factors alone – was adequate to detect caregivers with high grief. The results on the screening tool are shown in Table 3. This screening tool shared similar AUROC as the original logistic model (AUROC 0.76, 95% CI 0.70–0.82, p < 0.001). At the optimal cut-off of two or more key factors, the screening tool had a sensitivity of 0.91, a specificity of 0.42 and a false-negative rate of 0.09. In the presence of two or more key factors, the tool could capture a larger proportion of caregivers with high grief (25.5%) relative to the baseline prevalence of 18.0%, which lent support to the utility of this tool.

**Table 3:** Utility of the key factors in identifying caregivers with high grief in the derivation sample (*n* = 300). The optimal cut-off score is highlighted in bold

Cut-off based on the number of key factors	Sensitivity	Specificity	False-negative rate	Number of caregivers	Number of caregivers with high grief (%) ^a^
0 or more	1.00	0.00	0.00	300	54 (18.0)
1 or more	1.00	0.05	0.00	288	54 (18.8)
**2 or more** ^**b**^	0.91	0.42	0.09	192	49 (25.5)
3 or more	0.43	0.93	0.57	40	23 (57.5)
4	0.06	1.00	0.94	4	3 (75.0)
AUROC (95% CI)	0.76 (0.70–0.82) ^c^				

*AUROC* area under the receiver operating characteristics curve

^a^High caregiver grief was defined as MM-CGI > 175

^b^The optimal cut-off score was selected based on the balance between sensitivity and specificity, with a preference for slightly higher sensitivity as the tool is intended primarily for screening purposes

^c^*P* < 0.001

We repeated the analyses in our validation sample based on another 103 caregivers who completed the Chinese MM-CGI. This validation sample had a mean MM-CGI score of 144.1 (SD 28.0), and a 10.7% prevalence of high caregiver grief. The demographic information of this validation sample is shown in Additional file 3. The results from the validation sample (Table 4) remained consistent – the AUROC was similar at 0.77 (95% CI 0.66–0.88, *p* < 0.001), while the optimal cut-off was also at two or more key factors (sensitivity 1.00, specificity 0.40 and false-negative rate 0.00). In the presence of two or more key factors, the tool could similarly capture a larger proportion of caregivers with high grief (16.7%) relative to the baseline prevalence of 10.7% in the validation sample.

**Table 4:** Utility of the key factors in identifying caregivers with high grief in the validation sample (*n* = 103). The optimal cut-off score is highlighted in bold

Cut-off based on the number of key factors	Sensitivity	Specificity	False-negative rate	Number of caregivers	Number of caregivers with high grief (%) ^a^
0 or more	1.00	0.00	0.00	103	11 (10.7)
1 or more	1.00	0.02	0.00	101	11 (10.9)
**2 or more** ^**b**^	1.00	0.40	0.00	66	11 (16.7)
3 or more	0.46	0.85	0.55	19	5 (26.3)
4	0.09	1.00	0.91	1	1 (100.0)
AUROC (95% CI)	0.77 (0.66–0.88) ^c^				

*AUROC* area under the receiver operating characteristics curve

^a^High caregiver grief was defined as MM-CGI > 175

^b^The optimal cut-off score was selected based on the balance between sensitivity and specificity, with a preference for slightly higher sensitivity as the tool is intended primarily for screening purposes

^c^P < 0.001

## Discussion

This is the first study that has constructed a simple screening tool as a convenient approach to identify caregivers who may possibly have high grief. We identified four key factors of grief in family caregivers of PWD – stage of dementia, behavioral problems in PWD, spousal caregivers and caregivers who provide daily care. We then demonstrated that the number of key factors alone can be sufficient to identify those who may require further assessment for high grief – a cut-off of two or more key factors provides a screening strategy which has high sensitivity and low false-negative rate. Similar results were consistently replicated in our validation sample.

The four key factors of caregiver grief that we identified in this study are understandable in the context of dementia caregiving. With increasing severity of dementia (the first key factor), the experience of loss and grief can become more palpable to caregivers [12, 20, 21] – the ambiguous loss of the PWD may become more prominent while the physical death of the PWD is more closely anticipated by caregivers. On the other hand, behavioral problems in PWD (the second key factor) can dominate relationships and convey the impression that the person is but a vestige of what he used to be. Such drastic changes in personality, along with changes in communication and reciprocity, can stimulate strong reactions of grief and loss in caregivers [2]. Spousal caregivers (the third key factor) generally have longer and closer bonds with the PWD [3] and may experience more grief reaction with the loss of the emotional closeness. Meanwhile, caregivers who provide care on a daily basis (the fourth key factor) are more likely to be reminded of the loss of personhood each time they are in contact with the PWD. Even with the apparent loss of personhood in the PWD, many of the PWD can still have periods of lucidity where their “former” selves resurface [9]. Daily contact with such vacillation in lucidity can be emotionally destabilizing to the caregivers [9, 21, 22] as they have to grapple with the uncertainty of loss.

The simple screening tool that we derived can be used as an efficient way to identify caregivers who may require further assessment for high grief. As described in the introduction section above, it is possible for us to detect high grief using the MM-CGI or MM-CGI-Short-Form (MM-CGI-SF) [10, 11]. However, this will involve administering the scale to all caregivers and incurring additional resources in manpower and time, as well as increasing the burden of administration to the caregivers. Moreover, the level of caregiver grief is likely to change over time and will require reassessment with changing caregiving circumstances (such as worsening dementia severity, increasing behavioral problem in PWD or changing in the amount of caregiving provided by each caregiver). Having a screening tool, we can focus only on those who are more likely to benefit from further assessment using specific grief scales such as the MM-CGI or MM-CGI-SF [10, 11]. Based on the participants from this study by way of example (Tables 3 and 4), we only need to focus on caregivers with two or more key factors and hence only administer the grief scale to two-third of the caregivers (192 caregivers from our derivation sample of 300 participants, or 66 caregivers from our validation sample with 103 participants). In other words, we can safely rule out a considerable proportion of caregivers who are less likely to have high grief (one-third of the caregivers in our given examples) since the screening tool has low false-negatives rates. Potentially, such use of the screening tool can translate into huge savings in resources in health services which provide care to large volume of PWD.

**Fig. 1:**
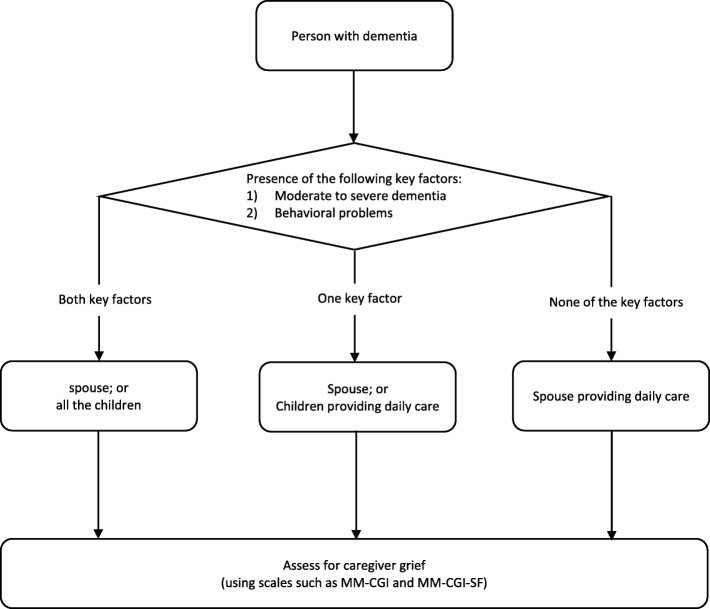
A proposed algorithm, based on the screening tool, that can be used to identify caregivers who may require further grief assessment using scales such as the Marwit-Meuser Caregiver Grief Inventory (MM-CGI) or the MM-CGI-Short Form (MM-CGI-SF)

We can further consolidate the screening tool into a simple algorithm that can be easily integrated into the workflow of routine health and social services which are involved in the care of PWD. As seen in Fig. 1, the algorithm begins by focusing on the PWD (which should fit naturally with most clinical and social care settings) and by identifying the two key factors in the PWD (moderate to severe dementia, or behavioral problems). Based on the presence of the two key factors in the PWD, it then directs practitioners to identify caregivers with specific characteristics (such as spouse, or children providing daily care to the PWD) for further grief evaluation, using scales such as the MM-CGI or MM-CGI-SF [10, 11] which have better specificity in confirming the presence of high grief. Due to its simplicity, this algorithm may be easily integrated as part of the routine workflow in health and social services, and hence can remove the need for dedicated staff to specifically perform this screening role. It can also be directly used by frontline staffs, on a regular basis, as a simple visual aid to alert them on the caregivers who may require from further referrals for grief assessments.

When caregivers are found to have high levels of grief, they may possibly benefit from interventions similar to what we often provide to those with difficulties related to post-death grief. Notably, grief-related interventions are distinct from those used to address caregiver burden in existing dementia services (where the focus is on reducing the perceived burden of caregivers and improving their coping skills; as guided by the transactional model of stress and coping) [23]. Grief-related interventions are more geared towards addressing the emotions, perceptions and behaviors related to losses [8, 13]. They may include facilitating caregivers to revisit the loss and its associated emotions [24, 25], reconstruct the meaning of the loss [26], find continual connection with the lost relationship [25, 27], involve the whole family in the grieving process [26, 28] and adjust to life changes following the loss [24, 25]. Ultimately, the goal of interventions is to realign the dyadic relationship [8, 29], to ensure that the caregiver can adapt to the losses and continue to maintain meaningful relationships with the PWD who may have changed due to the dementing processes.

Some limitations of the study should be noted. First, the participants were recruited from dementia clinics in tertiary hospitals and may not necessarily represent community-dwelling caregivers. Notwithstanding this limitation, the simple screening tool can be especially useful in settings similar to our recruitment centers, such as other memory clinics and dementia services, to identify caregivers with potentially high grief. Second, the proportion of spousal caregivers (11.7%) was relatively lower than that of children caregivers. However, this proportion is similar to the 16.0% reported in another nationally-representative study in Singapore [30] and hence is unlikely due to any sampling bias. Third, although the screening tool has high sensitivity, it may not be specific to caregiver grief and may potentially identify caregivers with other difficulties unrelated to grief, such those with high burden or depression. Hence, this new tool should not be used for diagnostic purposes (with those screened positive regarded as having high grief), but should trigger the need for further evaluation (using a scale such as MM-CGI or MM-CGI-SF) [10, 11] to verify the caregivers’ need for grief-related intervention. While the key factors from this study bear some semblance to the risk factors of caregiver burden, the factors associated with grief and burden are not necessarily synonymous. This is reflected in a recent study [13], which demonstrated the shared risk factors between grief and burden (such as behavioral problems in PWD; and high amount of caregiving duties), as well as the differences in risk factors between them (for example, spousal caregiver was associated with grief but not burden; and severe stage of dementia was more strongly associated with grief than burden).

## Conclusions

In conclusion, four key factors for high caregiver grief were identified in this study. They are consistent with current understanding on caregiver grief in the literature, and can be easily integrated into the workflow of routine services to screen for caregivers who are more likely to benefit from further grief-related assessment.

## Additional file

Additional file 1: Further details on the evaluation of the final regression model in its goodness of fit and discriminative value. (DOCX 37 kb)

Additional file 2: Results on the goodness of fit and discriminative value of the final regression model. (DOCX 38 kb)

Additional file 3: Demographic characteristics of the caregivers and persons with dementia in the separate validation sample (*n* = 103) (DOCX 35 kb)

## Abbreviations

CI: confidence interval
DSM-5: the fifth edition of Diagnostic and Statistical Manual of Mental Disorders
DSM-III-R: the revised third edition of Diagnostic and Statistical Manual of Mental Disorders
DSM-IV: the fourth edition of Diagnostic and Statistical Manual of Mental Disorders
MM-CGI: Marwit-Meuser Caregiver Grief Inventory
MM-CGI-SF: Marwit-Meuser Caregiver Grief Inventory-Short Form
PWD: persons with dementia
SD: standard deviation

## References

[1] Liew TM, Yeap BI, Koh GC, Gandhi M, Tan KS, Luo N, Yap P (2018). Detecting Predeath grief in family caregivers of persons with dementia: validity and utility of the Marwit-Meuser caregiver grief inventory in a multiethnic Asian population. Gerontologist.

[2] Feast A, Orrell M, Charlesworth G, Melunsky N, Poland F, Moniz-Cook E (2016). Behavioural and psychological symptoms in dementia and the challenges for family carers: systematic review. Br J Psychiatry.

[3] Pozzebon M, Douglas J, Ames D (2016). Spouses’ experience of living with a partner diagnosed with a dementia: a synthesis of the qualitative research. Int Psychogeriatr.

[4] Piiparinen R, Whitlatch CJ (2011). Existential loss as a determinant to well-being in the dementia caregiving dyad: a conceptual model. Dementia.

[5] Liew TM, Yap P, Luo N, Hia SB, Koh GC, Tai BC (2018). Detecting pre-death grief in family caregivers of persons with dementia: measurement equivalence of the mandarin-Chinese version of Marwit-Meuser caregiver grief inventory. BMC Geriatr.

[6] Chan D, Livingston G, Jones L, Sampson EL (2013). Grief reactions in dementia carers: a systematic review. Int J Geriatr Psychiatry.

[7] Walker RJ, Pomeroy EC (1997). The impact of anticipatory grief on caregivers of persons with Alzheimer's disease. Home Health Care Serv Q.

[8] Liew TM, Tai BC, Yap P, Koh GC. Comparing the effects of grief and burden on caregiver depression in dementia caregiving: a longitudinal path analysis over 2.5 years. J Am Med Dir Assoc. 2019. doi: 10.1016/j.jamda.2018.11.016. [Epub ahead of print] PubMed PMID: 30692034. 10.1016/j.jamda.2018.11.016 30692034

[9] Lindauer A, Harvath TA (2014). Pre-death grief in the context of dementia caregiving: a concept analysis. J Adv Nurs.

[10] Marwit SJ, Meuser TM (2002). Development and initial validation of an inventory to assess grief in caregivers of persons with Alzheimer's disease. Gerontologist.

[11] Marwit SJ, Meuser TM (2005). Development of a short form inventory to assess grief in caregivers of dementia patients. Death Stud.

[12] Liew TM (2016). Applicability of the pre-death grief concept to dementia family caregivers in Asia. Int J Geriatr Psychiatry.

[13] Liew TM, Tai BC, Yap P, Koh GC (2019). Contrasting the risk factors of grief and burden in caregivers of persons with dementia: multivariate analysis. Int J Geriatr Psychiatry.

[14] American Psychiatric Association (1987). Diagnostic and statistical manual of mental disorders: DSM-III-R.

[15] American Psychiatric Association (2013). Diagnostic and statistical manual of mental disorders: DSM-5.

[16] Forsell Y, Fratiglioni L, Grut M, Viitanen M, Winblad B (1992). Clinical staging of dementia in a population survey: comparison of DSM-III-R and the Washington University clinical dementia rating scale. Acta Psychiatr Scand.

[17] Juva K, Sulkava R, Erkinjuntti T, Ylikoski R, Valvanne J, Tilvis R (1994). Staging the severity of dementia: comparison of clinical (CDR, DSM-III-R), functional (ADL, IADL) and cognitive (MMSE) scales. Acta Neurol Scand.

[18] Grobbee DE, Hoes AW (2014). Clinical epidemiology.

[19] Hosmer DW, Lemeshow S, Sturdivant RX (2013). Applied logistic regression.

[20] Ott CH, Sanders S, Kelber ST (2007). Grief and personal growth experience of spouses and adult-child caregivers of individuals with Alzheimer's disease and related dementias. Gerontologist.

[21] Adams KB, Sanders S (2004). Alzheimer’s caregiver differences in experience of loss, grief reactions and depressive symptoms across stage of disease:a mixed-method analysis. Dementia.

[22] Furlini L (2001). The parent they knew and the “new” parent: daughters’ perceptions of dementia of the Alzheimer’s type. Home Health Care Serv Q.

[23] Lazarus RS, Folkman S (1984). Stress, appraisal, and coping.

[24] Stroebe M, Schut H (2010). The dual process model of coping with bereavement: a decade on. OMEGA – J Death Dying.

[25] J. William Worden PDA: Grief Counseling and Grief Therapy, Fourth Edition: A Handbook for the Mental Health Practitioner. New York: Springer Publishing Company 2008.

[26] Neimeyer RA (2014). The changing face of grief: contemporary directions in theory, research, and practice. Progress in Palliative Care.

[27] Klass D, Silverman PR, Nickman SL. Continuing bonds: new understandings of grief. New York: Taylor & Francis; 1996.

[28] Stroebe M, Schut H (2015). Family matters in bereavement: toward an integrative intra-interpersonal coping model. Perspect Psychol Sci.

[29] Kahana E, Young R, Blum DEBA (1990). Clarifying the caregiving paradigm: Challenges for the future. Aging and caregiving: Theory, research, and policy.

[30] Malhotra R, Chan A, Malhotra C, Ostbye T (2012). Validity and reliability of the caregiver reaction assessment scale among primary informal caregivers for older persons in Singapore. Aging Ment Health.

