# Supplementary material for: Development and validation of a simple screening tool for caregiver grief in dementia caregiving
Source: BMC Geriatr. 2019 Feb 27;19:54. doi: 10.1186/s12877-019-1070-x (PMC6391777; doi:10.1186/s12877-019-1070-x)
Supplement: Supplementary file 1 — Further details on the evaluation of the final regression model in its goodness of fit and discriminative value. (DOCX 37 kb) [file 12877_2019_1070_MOESM1_ESM.docx]

**Additional file 1.** Further details on the evaluation of the final regression model in its goodness of fit and discriminative value.

The goodness of fit of the final regression model was assessed using the Hosmer-Lemeshow test and the calibration plot. The discriminative value of the model was assessed by computing the area under the receiver operating characteristics curve (AUROC) of the predicted probabilities – an AUROC of 0.7–0.8 is considered acceptable, and more than 0.8 is considered excellent [1]. The discriminative value of each included variable in the model was also assessed using the Integrative Discrimination Improvement (IDI) [2] and Category-free Net Reclassification Improvement (cNRI) [3] indices – positive values in IDI and cNRI (with 95% CI that do not include the value of zero) indicate that a variable significantly improves the discriminative ability of the model.

**ADDITIONAL REFERENCES**

1. Hosmer DW, Lemeshow S, Sturdivant RX: **Applied logistic regression**, Third edition. edn. New Jersey: Wiley; 2013.

2. Pencina MJ, D'Agostino RB, Sr., D'Agostino RB, Jr., Vasan RS: **Evaluating the added predictive ability of a new marker: from area under the ROC curve to reclassification and beyond**. *Stat Med* 2008, **27**(2):157-172; discussion 207-112.

3. Pencina MJ, D'Agostino RB, Sr., Steyerberg EW: **Extensions of net reclassification improvement calculations to measure usefulness of new biomarkers**. *Stat Med* 2011, **30**(1):11-21.
