# Supplementary material for: Development and validation of a simple screening tool for caregiver grief in dementia caregiving
Source: BMC Geriatr. 2019 Feb 27;19:54. doi: 10.1186/s12877-019-1070-x (PMC6391777; doi:10.1186/s12877-019-1070-x)
Supplement: Supplementary file 2 — Results on the goodness of fit and discriminative value of the final regression model. (DOCX 38 kb) [file 12877_2019_1070_MOESM2_ESM.docx]

**Additional file 2.** Results on the goodness of fit and discriminative value of the final regression model.

The final regression model demonstrated a good fit in the Hosmer-Lemeshow test (p=0.718), with the calibration plot showing agreement between the predicted probability and observed frequency of high grief as shown below:

The four key variables in the final regression model also demonstrated discriminative values as shown in the table below. Notably, IDI and cNRI had positive values (with 95% CI that do not include the value of zero), which indicates the significance of each of the key variables in improving the discriminative ability of the model.

| Key factor included in the logistic regression | AUROC (95% CI) | IDI, % (95% CI) ^a^ | cNRI, % (95% CI) ^a^ |
| --- | --- | --- | --- |
| Best single key factor (S) | 0.63 (0.57, 0.70) | - | - |
| Best 2 key factors (S + B) | 0.69 (0.62, 0.76) | 4.2 (0.2, 13.0) | 30.5 (10.4, 52.9) |
| Best 3 key factors (S + B + D) | 0.73 (0.65, 0.80) | 1.9 (0.1, 5.8) | 32.2 (7.2, 60.8) |
| All 4 key factors (S + B + D + M) | 0.76 (0.69, 0.82) | 2.6 (0.3, 6.1) | 30.8 (-9.0, 43.9) |

AUROC, area under the receiver operating characteristics curve; 95% CI, 95% confidence interval; IDI, Integrated discrimination improvement; cNRI, Category-free Net reclassification improvement; HL, Hosmer-Lemeshow test; S, spousal relationship; B, behavioral problem in persons with dementia; D, daily caregiving; M, moderate to severe stage of dementia.

^a^ The 95% CI of IDI and NRI were estimated using the bootstrap method. The value is considered significant if the 95% CI does not include zero.
