# Supplementary material for: Development and validation of a simple screening tool for caregiver grief in dementia caregiving
Source: BMC Geriatr. 2019 Feb 27;19:54. doi: 10.1186/s12877-019-1070-x (PMC6391777; doi:10.1186/s12877-019-1070-x)
Supplement: Supplementary file 3 — Demographic characteristics of the caregivers and persons with dementia in the separate validation sample (n = 103) (DOCX 35 kb) [file 12877_2019_1070_MOESM3_ESM.docx]

**Additional file 3.** Demographic characteristics of the caregivers and persons with dementia in the separate validation sample (n=103)

| Variable | n (%) |
| --- | --- |
|  |  |
| **VARIABLES RELATED TO CAREGIVERS** | |
| Age, mean (SD) | 55.2 (9.3) |
| Female gender | 42 (40.8) |
| Ethnic |  |
| Chinese | 103 (100) |
| Malay | 0 (0) |
| Indian/Eurasian/Others | 0 (0) |
| Marital status |  |
| Married | 74 (71.8) |
| Single | 21 (20.4) |
| Widowed/Divorced/Separated | 8 (7.8) |
| Employment status |  |
| Working full-time | 47 (45.6) |
| Working part-time | 16 (15.5) |
| Not working | 40 (38.8) |
| Highest education |  |
| Secondary or below | 87 (84.5) |
| Tertiary | 16 (15.5) |
| Relationship with PWD |  |
| Child | 84 (81.6) |
| Spouse | 19 (18.5) |
| Staying with PWD | 70 (68.0) |
| Duration of caregiving in years, mean (SD) | 7.2 (6.6) |
| Providing daily caregiving | 74 (71.8) |
| Primary caregiving role | 65 (63.1) |
|  |  |
| **VARIABLES RELATED TO PWD** |  |
| Age, mean (SD) | 79.7 (8.3) |
| Female gender | 31 (30.1) |
| Duration of dementia diagnosis in years, mean (SD) | 4.3 (3.3) |
| Onset of dementia before 65 years | 15 (14.6) |
| Stage of dementia |  |
| Mild | 14 (13.6) |
| Moderate | 41 (39.8) |
| Severe | 48 (46.6) |
| Severe behavioral problem | 5 (4.9) |

SD: standard deviation; PWD: persons with dementia; MM-CGI: Marwit-Meuser Caregiver Grief Inventory.
